# Generalization through similarity: motif discourse in the discovery and elaboration of zinc finger proteins

**DOI:** 10.1186/1747-5333-2-5

**Published:** 2007-10-03

**Authors:** Celeste Michelle Condit, L Bruce Railsback

**Affiliations:** 1 Department of Speech Communication, 110 Terrell Hall, University of Georgia, Athens, GA, USA; 2 Department of Geology, University of Georgia, Athens, GA, USA

## Abstract

**Background:**

Biological organisms and their components are better conceived within categories based on similarity rather than on identity. Biologists routinely operate with similarity-based concepts such as "model organism" and "motif." There has been little exploration of the characteristics of the similarity-based categories that exist in biology. This study uses the case of the discovery and classification of zinc finger proteins to explore how biological categories based in similarity are represented.

**Results:**

The existence of a category of "zinc finger proteins" was based in 1) a lumpy gradient of similarity, 2) a link between function and structure, 3) establishment of a range of appearance across systems and organisms, and 4) an evolutionary locus as a historically based common-ground.

**Conclusion:**

More systematic application of the idea of similarity-based categorization might eliminate the assumption that biological characteristics can only contribute to narrow categorization of humans. It also raises possibilities for refining data-driven exploration efforts.

## Introduction

Biological beings can be understood as physical matter arranged in particular sets of patterns that create characteristics different from other physical matter. These patterns have been described as including form replication, developmental differentiation, boundary maintenance, nested hierarchical order, negative entropy, and representationally guided movement, among others [Ch. 4, especially Table [1]]. As Ernst Mayr observed, a notable consequence of these unique characteristics has been that scientific efforts to understand biological beings have required different research assumptions and processes than other scientific endeavors, most notably classical physics [2]. One of the key changes in scientific assumptions wrought by the blossoming of biology has been a shift from identity to similarity as a basis for classification and generalization. Unlike fluorine atoms or muons, which have historically been presumed to be identical within their classes, no two biological beings are exactly the same. Biologists therefore routinely use similarity-based concepts, such as "model organism" and "motif", both as research tools and as modes of explanation.

Despite the widespread use in biology of categories based on similarities rather than identities, the idea that similarity provides the central basis for classification has not yet been thoroughly integrated into higher order conceptions of the study of biology, or into its philosophy. The overwhelming majority of the biologists with whom we have interacted have articulated as their over-arching rationale the philosophy of science based on classical physics, which depends on the notion of identity. Further consideration of the nature of similarity-based classification is thus warranted to facilitate its integration into practice and a more appropriate general philosophy of biology. This essay will begin with a brief explanation of the difference between a "classical" physics-based philosophy of science, with its dependence on identity, and a biologically-based approach in which similarity is critical. It will then examine the case study of the discovery of zinc finger proteins as an illustration of the logic of similarity-based classification in biological discovery. The essay then will discuss two arenas in which a principled application of the biologically-based philosophy of science has potentially valuable implications. These are the use of biologically-based arguments in social discussions of the nature of human groups and the biologically-derived approach for data-driven discovery methods.

## Biology and the philosophy of science

The understanding of the nature of physical matter developed by classical physics has long been superceded by quantum physics and relativity theory. Nonetheless, the philosophy of science that was developed as a consequence of the early successes in physics, and which was most famously codified by Karl Popper, [3] continues to hold sway as the default account of "what science is" (outside of some sets of experts in science studies). Perhaps the endurance of the classic account is merely a product of an effect of primogeniture – science in an important sense got its most visible "start" through its notable successes in this early period, so the sense that "this is what science must be" has a strong presumption. Perhaps, however, the classical account endures merely or also because it coincides with the simple linear cause-effect grammars built into natural human language systems [[4], pp. 112, 172].

Whatever the causes for its survival, the classical account of how science works and its attendant assumptions about the nature of being have been shown to be, at the least, highly incomplete [5]. These accounts tend to put almost exclusive weight on "crucial experiments", which are hypothesis-driven manipulations of the natural world, the results of which definitely discredit a particular theoretical formulation. According to the most narrow formulations, the hypotheses must be in quantitative form. For the more lenient, results may actually be said to support one theoretical account in contrast to another, rather than merely falsify a hypothesis or even a theory.

Regardless of the formulation one applies, and although crucial experiment is indeed an essential component of scientific practice, experimentation accounts for only a small proportion of the research efforts in the biological sciences and elsewhere (from geology to empirically driven materials science to human social sciences). Systematic observation plays a larger role (though biologists and earth scientists routinely obscure this fact by mis-labeling their systematic observations as "experiments"). For example, neither Darwin's theory of evolution nor Watson and Crick's account of the structure of the double helix were derived from crucial experiments. Watson and Crick did rely heavily on the observational data garnered by others, and evolutionary theory rests on a vast network of observations and sub-crucial experiments. Furthermore, Popper's prescription that science can only be falsification is unworkable. In order to build the inter-related superstructures that characterize all modern science, an enormous number of facts and theories must be accepted (albeit always provisionally). The processes by which these facts and theories are accepted includes more observation, induction, and logical and mathematical analysis than falsification (though falsification plays a pivotal role in many cases).

The view of both physical matter and of scientific practice that was developed through the research efforts of classical physics was thus errant in several respects. This essay addresses the classical assumption of identity-based categorization. The classical view held that phenomena had properties that were universal across time and space. As Franz Wilzeck put it,

matter is built up from vast numbers of copies of a few fundamental components (such as electrons, quarks, photons, and gluons). The properties of these elementary building blocks are always and everywhere the same – universal [6].

This universality and self-identical property meant that mathematical *laws *could describe the behavior of objects based on an invariant set of properties. On this view, all fluorine atoms were identical, which operationally meant that each could be expected to behave in exactly the same way under any given set of circumstances.

This set of assumptions was never actually correct. Oxygen and carbon atoms, for example, have different isotopes, and specific isotopic forms have different implications in both geologic and biological frameworks [7]. Moreover, as with all the assumptions of classical physics, the abstract theory never applied exactly to actual objects. One can describe, in the abstract, what the forces on an atom should look like, but one can never actually compute those forces for a particular atom (both because of the Heisenberg principle and for more practical, mundane reasons). Additionally, when applying physical laws to particular objects, a world of exceptions and exclusions have to be countenanced (e.g. ignore friction, discount resistance, pretend space is an absolute vacuum, etc.). In other words, the presumption of identity was always enabled only by the fact that classical physics dealt almost exclusively with large sets of atoms, across which the variations in individuals were masked. As physicists have begun to gain the capacity to examine atoms on the individual level, indications have began to appear that the inevitable small variations among atoms (arising from contingencies such as recent histories of relationships to other atoms that might "deform" them in a specific fashion, albeit temporarily) make even atoms of a given category "similar" rather than "identical" to one another [8]. Thus, even with regard to atoms, classical physics's assumption that classes of items were universally identical probably does not hold.

The tendency to treat categories as though they were collections of identical elements has been attributed to the characteristics of language [9]. Words are discrete units that appear in common sense to be processed as self-identical. That is, our common-sense intuition leads us to presume that variations in pronunciation or typography are "washed out" because words "refer" to stable, uniform "ideas". Recent research using neuro-imaging techniques has produced distributed neural network theories of language processing that indicate this common-sense feeling is false [10]. Because words are processed in streams that activate different portions of neural networks in which particular words are embedded, each activation is an activation of a somewhat different portion of the neural network. Pronunciation and typography may contribute to this variation to different extents in different congeries [11]. The falsity of our common-sense notion, however, has not abrogated the pervasiveness with which we apply it. On this account, the attribution of universality and category identity to the objects of classical physics is simply a particularly visible and influential example of a more pervasive error promoted by the characteristics of human language processing.

It is with this observation, that all linguistic categories represent variable constructions rather than permanent, universal ideas, that most critiques in "science studies" end. This basic insight forms much of the impetus for deconstructive treatments of science (though the formulations may vary, emphasizing for example, categories as binary systems [12], as metaphors [13], or as other linguistic resources [14]). Such a stopping place, however, ironically replays the problem it alleges of classical physics; it assumes identity among the similarities across different categories. That is, it assumes that the matter of biological beings, of non-biological physical matter, and of symbolic processes are for all intents and purposes identical, and sometimes, further, that they are a product solely of the shared linguistic processes applied to all. Instead, it is useful and materially appropriate to posit different "modes" of being – physical, biological, symbolic, and perhaps artificial life/intelligence – which constitute substantially different arrangements of physical matter, and which therefore manifest different patterns of similarities within the categories humans constitute to discuss and manipulate them [1].

If some basic patterns in the similarities that underlie biological organisms can be identified, then perhaps that information can provide useful guidance not only with regard to the broad issues of the philosophy of biology but also with regard to both the practice of biology and the integration of a biological world view into our social concepts. A case study of a classic, well-accepted, and important biological discovery provides a place to begin such an exploration.

## Zinc finger proteins

Both when Miller, McLachlan & Klug [15] reported their discovery of zinc finger proteins and when Laity, Lee, and Wright [16] fifteen years later published a much broader codification of zinc finger proteins as a class, four fundamental principles for making judgments of similarity-based generalizations in biology were evident in their reports. These included 1) a lumpy gradient of similarity, 2) a link between function and structure, 3) establishment of a range of appearance across systems and organisms, and 4) an evolutionary locus as a historically based common-ground. Awareness of these facets of similarity-based generalization may enhance the ability to construct methodologies and informatic systems that can, among other innovations, better identify candidate genetic and other motifs that play causative roles in disease.

### Gradients of similarity

When Miller, McLachlan & Klug first identified what they called the "Repetitive zinc-binding domains in the protein transcription factor IIIA from *Xenopus *oocytes," they introduced the rationale for the paper on the grounds that they had found "a remarkable repeating structure within the protein" [[15], p. 1609]. This repeating structure was not, however, a set of identical amino acids that recurred in series. Rather, it was a set of "roughly periodical groupings" (p. 1610).

By the time of Miller, McLachlan & King's discovery, the concept of the "motif" had already become relatively common in genetics. A "motif" was identified as a sequence of DNA bases that were highly similar across systems or organisms. That is, the string of base pairs was not identical from one gene to another, or from one organism to another. Rather, there existed a small number of "highly conserved sequences" embedded within a larger number of sequences with more variation, but which shared frequencies at a higher level than mere chance. Even the "highly conserved sequences" were not necessarily identical, but they were the same across *most *genes or organisms within which the "motif" was deemed to appear.

A motif was thus tacitly understood to be identifiable by a gradient of similarity – a highly conserved (nearly but not quite identical) region embedded in a region of lesser conserved (but not random) base sequences. These gradients were not uniform, but rather lumpy. That is, a biologically based similarity may not manifest a smooth slope of increasing and decreasing similarity; instead, it may feature several zones with higher concentrations of similarity. This distinctive form is a product of evolutionary forces at many scales. At the genetic scale, highly conserved sequences appear where an important function reduces the number of viable mutations. As one moves away from these critical spots, a greater number of mutations are tolerable. But the multiple folds of most proteins mean that any given protein features not just one, but several critical spots (usually spots where the protein contacts other biological components) that tolerate little variation, surrounded by increasingly distant spots that tolerate a wider range of variations.

**Figure 1:**
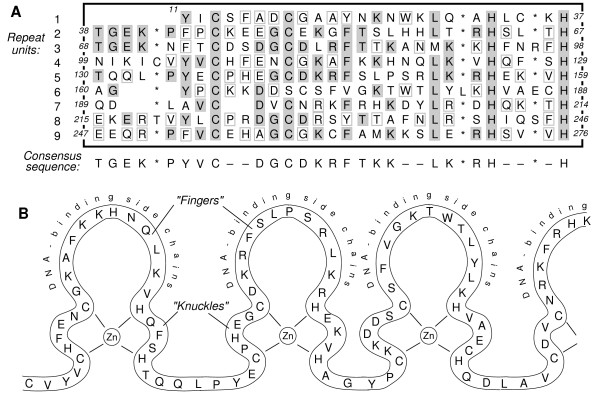
**Amino acids in zinc fingers**. A: In large dark rectangle, sequence of amino acids 11 to 276 in transcription factor IIIA of *Xenotopus laevis *oocytes, aligned in nine rows to show extent of repetition of repeat units. Letters below rectangle indicate consensus sequence derived by Miller, McLachlan, and Klug (1985); dashes in consensus sequence indicate positions of no consensus. Shaded letters highlight acids that match the consensus sequence; letters in small gray rectangles indicate acids that do not match the consensus sequence but match at least one other acid in that position; unshaded letters match neither the consensus sequence nor any other acid. Blanks indicate gaps in the alignment, with the gaps positioned to maximize match of surrounding acids with the consensus sequence. Asterisks do not represent acids but indicate positions at which "insertions" commonly occur. Adapted from Figure 3 of Miller, McLachlan, and Klug (1985). B: Model of the arrangement of amino acids 103 to 204 in transcription factor IIIA of *Xenotopus laevis *oocytes. Three complete "zinc fingers" are shown. Adapted from Figure 4 of Miller, McLachlan, and Klug (1985). Note that the amino acids that most consistently match the consensus sequence in A are those that serve as ligands with zinc in B.

The conception of the "motif" provided a mode of generalization based on a specific type of pattern recurrence, not identity. Thus, in characterizing a set of proteins, Miller, McClachlan & Klug had ready-to-hand a word from the study of DNA describing a type of similarity that fit the "repetitive zinc-binding domains" that they were seeking to understand. They described their proteins as showing "an exceptionally strong and regular pattern of 30-residue repeats in the sequences with four repetitions evident in the first half of the molecule (residues 13–156) and two more clear repeats in the second half (residues 223–276)." To the eye, this pattern looks significant. It doesn't look like a random scatter (Fig. 1a). Establishing the meaningfulness of the apparent patterns in a fashion that exceeds intuition was not, however, a straightforward manner.

The reasonable approach was to assess the likelihood of such patterns occurring by chance as opposed to occurring as the product of some underlying causal driver. For such purposes, statistical methods are commonly used to compare "expected" values in unbiased conditions to "observed" values. However, as is typical in biology, it was not trivially obvious what statistical tests were appropriate to the case. Consequently, in the appendix on "Materials and Methods" the authors addressed in three different ways (for two components of their data) the potential approaches to establishing the statistical significance of the patterns they had observed.

Two facets of their analysis are of interest for present purposes. First, one of the approaches they employed took some advantage of the gradients of similarity that occur in biology, as it assigned weights to scores "which die away exponentially with distance from the center of each window." They noted, however, that "this newer method is more suitable for dealing with gaps, but is less susceptible to statistical analysis." This tension between the available statistical methods of the time and the nature of similarity gradients illustrates some enduring features that will be addressed in the discussion of data-driven methods below.

The second issue of interest is the argument they made for the similarity of the 344-residue sequences. We want to be clear that we are not criticizing their argument, because we think it was a completely reasonable one. Instead, we are merely pointing out that there was no self-evident test that could or should have been used in the case. The authors set a threshold value based on expected appearance, which was further based on the reasonable (but not necessary) assumption of their alignment of 344 selected residues as the relevant comparison basis. They then noted that the value was "exceeded not once, but 359 times in the natural sequence and showed that there are many significant repetitions." As was standard for that era, no estimate of the number of comparisons involved was given. 359 matches certainly sounds like a lot – even overwhelming. However, because chance occurrences cumulate with multiple testing runs, and because the number of potential testing runs in many biological cases quickly becomes quite large, one of the things biologists have had to become sensitized to is reporting statistics in terms of adjustments for such accumulations. Statistical analysis, however, offers no definitive criteria for establishing appropriate parameters for the numbers involved (not only the choice of how many residues 'out' the analysis should include but also which comparisons are salient and which extraneous and how large a correction is required). This "multiple testing" problem is endemic to biological phenomena because of their complexity. This too will be addressed in more detail below.

Miller, McClachlan & Klug's identification of the motif was not, however, grounded solely in the statistical argument about likely co-occurrence of particular sequences. It was further bolstered by the ability to situate the similar elements within a gradient of increasing/decreasing similarity. Thus, the authors were able to focus on greater levels of similarity within some units than others, noting that "it can be seen that units 5, 9 and 2, in that order, are clearly the most typical members of the family, and are very like one another" (p. 1611). By abstracting above the level of the individual base pair sequences, they were able to identify a "characteristic Cys-Cys-His-His consensus motif." The case for the existence of the zinc finger as a biological type with predictable properties and actions was also dependent on the ability to specify a function for the sequence.

### A link between form and function

An extensive debate has been conducted over whether the existence of an identifiable form is an infallible sign of the existence of a function for that form [17,18]. We agree with those who suggest that identifiable forms may endure in a species for some time beyond their evolved functions. Nonetheless, because form so pervasively is evolutionarily a product of function among biological beings, the ability to link a form to a biological function stands as evidence that the form should be understood as a distinctive biological entity. Miller, McLachlan, and Klug's paper is convincing (and significant) in large measure because they were able to link the self-similar entity of the Cys-Cys-His-His motif to a potential function. As they pointed out, the structure of the sequence appears to resemble "fingers" that can act as "DNA-binding fingers, linked by (flexible?) joints" (p. 1613) (Fig. 1b). They were able to note that "a structure of this kind would explain how a relatively small protein of 40 K can bind to a long stretch of double-helical DNA" (p. 1613).

The ability to identify a function for a gradient of similarity stands as a warrant for treating that form as an entity in biology because functionality is a distinctive property of biological beings. Non-biological physical entities appear in a given form solely as a consequence of the manifestation of the balance of immediately present physical forces and the accumulated history of these forces as they have interacted with the particular physical entity (that is with a set of particular atoms or a substantial subset thereof). For example, a newly-precipitated crystal has a form dictated by the interactions of the atoms within the solution from which it precipitates, even though it will resemble other crystals made from the same categories of atoms. In contrast, biological entities appear in their characteristic forms because a similar prior form included a template for that form, which was enabled to assemble the necessary physical matter to re-construct the form from the surrounding environment. This process is called reproduction or replication, and the term "function" is used to describe any set of interactions that increases reproducibility. The vocabulary of "function" is necessary for describing biology and central to its study, but not for the study of naturally occurring non-biological physical matter, because this reproducibility is a unique hallmark of biological organisms.

Asserting that biological entities have unique hallmarks discomforts people who are devoted to defending materialist or physicalist accounts of biology against "vitalism". But the assertion of distinctive qualities to matter arranged in biological forms does not require or entail belief in any mysterious spirits. The assembly of the template that allows form reproduction follows physical laws, and the re-assembly of the "offspring" form also follows physical laws, and both are possible only under a particular range of physical conditions. But the physical arrangements that fall in the category of "biological organisms" *do *have a form that can *only *be produced given the presence of the template, and this is not true of non-biological physical matter. Liquids and fluids have no characteristic form, and even solids assume a form that has no linear causal relation to the existence of a similar form of a prior object. The rounded pebble became a rounded pebble instead of a jagged hunk of rock because it tumbled in a stream, not because an older rounded pebble passed on a template that contained a code (i.e. a complex set of physical interactions) that would re-produce that form within a range of circumstances. The biological template may be nothing but a bunch of physical matter arranged in a specific and complex fashion, but that arrangement *does *produce distinctive characteristics that physical matter lacking such an arrangement does not manifest.

Thus, although biological forms can be said to be a product of the cumulative balance of physical forces, they are a product not merely of immediate forces upon a particular set of atoms, and not merely a product of the accumulated forces upon that subset of atoms, but also a product of the way in which physical forces have been distributed over time *in a specific lineage of forms*. Biological forms are thus not "other than" physical matter, but rather a unique subset of physical matter, whose uniqueness requires explanations not required for other types of matter.

Physical forms that can be reproduced through time appear at heightened frequencies so that reproducibility of form becomes a physical feature that distinctively identifies biological forms. Since functions are sub-requirements of reproducibility and this is distinctive of biology, decisions about whether two things belong to the same category reasonably require not merely the statistical recurrence of a form, but the form-function pairing.

### Range of generality

If one were to find a biological phenomenon that appeared in only one place in only one organism, this would be a strange biological phenomenon indeed. This is because, as far as we can tell, all life on Earth has evolved from a common source. Thus, typically, to identify the nature of a biological entity requires identification of the range of systems and organisms across which it occurs (in similar, though not identical, forms). In their original article, Miller, McLachlan, and Klug began to chart these boundaries. They searched for the protein within a large number of zinc enzymes and metallothioneins and reported failure to find the motif there (p. 1613). They also speculated that it "would also not be surprising if the same 30-residue units were later found to occur in varying numbers in other related gene control proteins" (1611). They were correct, although as is generally the case in biology, the more types of organisms and systems that are investigated, the broader the degradation of similarity, so that the form of the motif they identified would become just one concentration of similarity within a broader class.

### Evolutionary locus

The identity of a biological phenomenon is manifested as a gradient of similarity (usually genetic or structural) that can be linked to a biological function and whose existence and boundaries can be traced across systems and organisms. Ultimately, however, a core part of the explanation of a biological phenomenon is its evolution. A thing that had not evolved could not be a biological entity (biological entities only exist as a consequence of the template-enabled lineage of forms). Consequently, in their effort to identify this new biological entity – the zinc finger protein – Miller, McLachlan, and Klug showed that this entity could fit within evolutionary logics. They noted that "the evolutionary advantages of a repeating design are probably much the same as those in many other linear proteins" (p. 1611). They accounted for the distinctive form of the protein set by suggesting that "Probably a single functional unit that binds to a half-turn of DNA was once evolved, and then became used in much more subtle and specialized ways when a large number of similar units were joined in series" (p. 1611). They even speculated about a potential role in the pre-DNA "RNA world" for features of the unit. Although this level of deep evolutionary speculation is somewhat unusual for a short, technically focused paper, such an exploration of evolutionary fit is a logical basis for grounding the existence and identity of the new class.

### The zinc finger motif 15 years after its discovery

In a review article published in *Current Opinion in Structural Biology*, Laity, Lee, & Wright [16] summarized the insights produced by the explosion of research enhanced by Miller et al.'s identification of the biological entity "zinc finger proteins." They noted of these proteins that "their functions are extraordinarily diverse," and that "their structures are as diverse as their functions" (p. 39). In a world dominated by identity-based understandings of the classes of beings, it would not have been possible to have characterized this extraordinary range of forms and functions as a single entity ("zinc finger proteins"). However, the essay by Laity et al. made evident the way in which understanding this gradient of similarity, its functions, and its dispersion had contributed to "novel insights into mechanisms of DNA binding" and of transcriptional regulation (p. 39). Indeed, well before the review was published, the zinc finger motif had become a model of motif structure in introductory genetics textbooks [19].

Like the original article, the account of zinc fingers that Laity, Lee, & Wright provided was an account of lumpy gradients of similarity. At the center of the now expanded gradient stands the "classic" Cys_2_His_2 _zinc finger identified by Miller et al. The review article provided new descriptions of the basis of similarity that had been gained by cross-organismal explorations. For example, it noted that "approximately half of the known Cys_2_His_2 _zinc finger proteins contain a highly conserved linker of sequence TGEKP" (p. 39). But the article also summarized the charting of the further edges where similarity in specific sequences decreases, and similarity in one core element (the appearance of zinc) came to dominate, including the DM motif, the NC protein's "zinc knuckles," and zinc-based elements in RNA polymerase and ribosomal proteins.

The review article, like the original article, also anchored the identity of the zinc binding proteins in functions. Along with the expansion in form, the range of functions for the motif had also expanded. Not only had more knowledge been gained about the nucleic acid binding of the "family" of proteins, but the "superfamily" of proteins was now understood to play crucial roles in some protein-protein interactions. Like the original article, the review article also attended to the now much better understood range of the motif across biological systems. Here, however, an important tension was revealed. As the breadth of the motif's applicability grew, and its variability in structure and function became more evident, was there some point at which identifying this phenomenon as a single biological class became untenable? The shift from the use of "family" in the original article to the use of "superfamily" in the second article indicated this tension.

**Figure 2:**
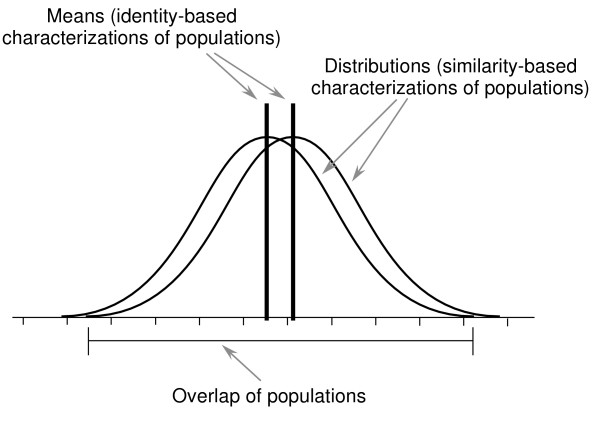
**Characterization of populations**. Schematic illustration of two populations in terms of distributions of some measurable parameter and in terms of the mean values for those distributions. Similarity-based characterization of these populations focuses on the distributions, whereas identity-based characterization focuses on the means and treats the distribution as noise or as aberrant values.

The authors further signaled this tension and responded to it in their concluding paragraph, saying "it is notable that recently determined structures of several previously uncharacterized zinc finger domains show that they are built on common structural cores, first seen in DNA-binding zinc fingers (the Cys_2_His_2 _motif [Figure 1a], GATA-1 [Figure 2a] and the β-ribbon zinc finger motif of TFHS [Figure [3b])" (p. 44). The coherence of the class was preserved by replacing a single center-point of the gradient of similarity with several different points of high local similarity (thus replaying across organismal systems the lumpy texture of the pattern of similarity that was first observed within the original DNA sequence set). Given the strong establishment of the forms and their functions, the review authors no longer seemed to feel the need to establish the potential evolutionary basis for the class. As forms and functions become taken-for-granted, explicit arguments indicating the consonance of the forms with evolutionary logics may appear obvious.

## The power of similarity-based generalization in biology and beyond

The processes involved in generalizing are quite different when one defines fluorine as any atom with 9 protons as compared to when one defines a zinc finger protein as "any small, functional, independently folded domain that requires coordination of one or more zinc ions to stabilize its structure" [[16], p. 39]. It might be argued that the former type of generalization is more appealing for a variety of reasons (and perhaps this is why it endures, even if it is widely inapplicable). It is more simple, and therefore more easily characterized as "elegant." It also seems to offer more power. If all fluorine atoms look exactly alike and respond exactly alike, then knowing a few things about fluorine enables one increased control of fluorine atoms in general. In contrast, the motif-based form of generalization common to biology can appear complex, which is often interpreted as "messy" and thus as inelegant. More importantly, motif-based generalization cannot promise the simplicity and directness of power and control of the identity-based model. As things diverge across a gradient of similarity, it is not clear which properties are shared where and therefore which interventions will work how. The failure of more than two thirds of drugs successfully tested in animals to also test successfully in human beings illuminates the serious limitations and costs associated with such forms of generalization [20].

In spite, however, of what seem to be taken as the appealing aesthetic qualities and power of the identity-based model of generalization, in biology the similarity-based model is simply required. This is because biological beings are, indeed, evolved through time in families. As evolution proceeds, systems diverge in forms and functions. No two beings are exactly alike, and every being is related to some degree. There is, therefore, truly no biological generality that is based on identity. The next question, however, is whether there are patterns of similarity within biological beings that have some commonality.

This study offers a tentative answer of "yes" to that question. The characteristic patterns of similarity in biological organisms include gradients of similarity (with lumpy regions of greater and lesser similarity), links between function and formal similarity, ranges of generality, and an evolutionary locus. However, additional research on different discoveries in other articles presenting well-established biological discoveries would be appropriate to explore the generality and sufficiency of these four elements. In the interim, it seems worth elaborating the substantive implications of such a framework.

### Social implications

The similarity-based nature of biological being has important implications for the role that conclusions from biology currently play in social debates over issues such as gender identity, racial differences, and individual susceptibility to mental illness or drug and alcohol abuse. Most often in such arguments, "biological causes" have come to be aligned with putatively universal patterns of form and behavior (via identity-based reasoning). For example, it is assumed that if there is a chromosomal difference between men and women then "men" must be a self-identical category and "women" a distinctly different, yet also self-identical category, each of which are identified by a set of particular behaviors different from the behaviors of the "opposing" category. Culture is then cast against biology in these arguments, with culture assuming the putative role of the source of all human variation.

An understanding of biological beings as entities that share ranges of similarities rather than identical properties re-orients this long-standing and portentous social argument. There is no one thing that is "the Y chromosome." Thousands of polymorphisms have been identified in the human Y chromosome (over 250 of which are currently reasonably well established), and many of these versions of this chromosome predispose its bearers toward *different *ranges of physical characteristics and behaviors [21]. While the average of all "Y" chromosomes (as modified by the literally million-fold genetic backgrounds with which they interact) may indeed produce a different "average" range of predispositions than that manifested by the "average" of all XX chromosome-bearers, the choice to identify that abstract "average" as the fixed identity of the category is a manifestation of identity thinking (the mis-begottten logic is that all XX's must be the same, so let's assume that the "average" is somehow the ideal; Figure 2). As Hyde & Linn's extensive survey of sex difference research has recently shown, in all cases but two (activity levels and aggression) these average differences between people of the two gender categories fell within the usual statistical standards for small or trivial effect size, while the ranges within both the groups "women" and "men" are much larger [22]. The fixation on a putative identity-basis for these two categories blinds one to the real variation among living bodies, and indeed grounds the creation of normative claims that seek to suppress that variation and even stigmatize and punish those who don't inhabit the respective "means". Focusing instead on gradients of similarities does not require one to ignore completely a difference of means, but it does require one to give appropriate attention to the range of the gradients. A similar analysis applies to racialized groupings, sexual orientation, or groupings by (dis)abilities.

A similarity-based understanding of biological classes thus allows a re-orientation of the on-going social argument about the causation of human characteristics, so that both biology and culture are recognized as sources of variation. At the least, however, the re-orientation requires recognition that because no two biological beings are identical, biological research cannot warrant claims to universal forms. The reorientation of our thought processes away from identity and toward expanded gradients of similarity that is required by the nature of biological beings has yet to be fully assimilated not only into our social discussions, but also into biological practices. The case of data-driven discovery provides a practical set of examples.

### Similarity and data-driven discovery

Currently, a large wave of what is often called "data-driven" science (as opposed to hypothesis-driven experiment) has come to genetic research as well as other disciplines. Although its appropriateness has sometimes been challenged, [23] the approach is grounded both in the availability of enormous piles of data in the form of DNA, mRNA, and protein sequences and also in necessity arising from the constitution of biological features through complexes of interacting networks that exhibit gradients of patterned variation across the biological domain. An enormous array of approaches are available for data-driven research, but a key thrust of these techniques is the use of computer programs to probe for statistical associations, either across organisms in the search for already understood analogues or between a nucleotide sequence and data about outcome characteristics pursuant to the search for new causal relationships.

Similarity-driven categorization principles offer at least three areas for potential improvement of data-driven research approaches. First, existing approaches to data-mining do not appear to take maximal advantage of the information available in biological systems. Second, the failure to apply regularly a logic of similarity has produced a substantial amount of error and waste in biological research, some of which might be avoidable. Third, the characteristics of biological similarity might provide some guidance about verification standards to be applied to data-driven discoveries.

#### Use more information

The data-mining approaches with which we are familiar are adapted to an identity-based logic rather than a logic based on gradients of similarity. They search for a statistical association between two variables (e.g. a stretch of DNA and a disease). While the identification of an association is based on probabilities, rather than a 100% match, there are no adjustments built into search algorithms that might give higher weight to patterned variations (i.e. "lumpiness'). One anonymous reviewer confirmed this tendency when s/he wrote with regard to an earlier draft of this paper, "The same Cys-Cys-His-His motif would have been apparent if the 'gradient' was flat (i.e. all members could have been equally like/unlike all other members except in the key regions of the CCHH motif)." While a true statement, this comment says that ignoring some of the available information in the structure of biological organisms doesn't matter. But since the gradient was not flat, and arguably many if not most biological gradients will not be flat, then to proceed with such an assumption is to ignore information that might be helpful. Depending on the object of study and the configuration of the mining technique, the surrounding lumps of similarity may appear as noise that obscures the highly similar component or as a false signal that unduly amplifies it, but neither is desirable. Data-mining programs sensitive to lumpy gradients of similarity would presumably be more accurate at identifying biologically significant phenomena. The use of sliding windows that weight similarity across distance (as employed by Miller, McLachlan, & King) seems to illustrate a potential track, although it is not yet calibrated to the recurrent "lumpiness" of gradients of similarity. Often, the patterns of interest will not be similarity merely within an individual segment of DNA, but rather similarity structures that occur across populations or through species trees.

#### Waste less, be wrong less often

The second area of opportunity relates to the error and waste that has occurred in data-driven discovery due to the failure to attend to similarity-based relations. A large number of scientific – and then journalistic – articles across the past three decades have claimed to find "a gene for" a variety of conditions, especially socially freighted conditions such as schizophrenia, alcoholism, brain size, drug use, and nurturing. Later studies repeatedly have revealed that the original results were statistical anomalies or due to population structure. In the case of alcoholism, for example, Conrad and Weinberg [24] have shown three different waves of announcements of the genetic roots of alcoholism. The strongest of the candidates for a causal input of genetics to alcoholism produced several contradictory results, and the results were eventually revealed to be highly sensitive to population structure [25].

These errors, and the attendant wastage, are a product of identity-based assumptions. Researchers went about their efforts with the assumption that all humans were the same except in a single causative allele for a condition. This is not to say that this assumption was a conscious, personal driver of their decision-making. The errant assumption may have rested within the funding agencies that called for such research. Or the driving force may have been the desire to test the simplest possibilities first, or to get the largest effects from the available data. Nonetheless, because the assumption is false, and humans come with a vast number of alleles that are arrayed in gradients of similarity based on ancestry, the statistical tests used picked up the ancestry or mere chance rather than causative alleles. Because they neglected to account for the similarity-based nature of the phenomenon they were looking at, the researchers then reported ancestry-derived or chance-derived associations as though they were causative alleles, and even if a causative allele was detected, they had no way to separate it from noise or background structure.

Identity-based assumptions have also encouraged persistence of the single-gene causation model well past any reasonable point. For example, an editorial attempting to adjudicate between a study showing a link between alcoholism and the DRD2 receptor and a study showing no link published in the same issue attempted to moderate the dispute by describing the allele in question as a "modifying" gene to an as-yet-undiscovered causative gene. The more reasonable conclusion is that there would be no single "alcoholism gene," but rather many alleles each of which make some relatively modest contribution in some genetic backgrounds and some environments [26]. If researchers had been thinking of biology in gradients of similarity, rather than as singular identities, such quests would not have appeared so promising, and researchers would not have been so likely to misinterpret noise and population structure as causative alleles.

Although these experiences have increased caution in these specific areas, especially leading to more rigorous controls for multiple testing, a more widely shared general understanding of the underlying similarity-based characteristics of biological phenomenon can help to reduce the likelihood of similar tendencies in other undertakings. It might also be possible to design verification procedures that help to weed out spurious findings.

#### Verifying data-driven findings

The embarrassing errors and wastage of data-driven approaches to finding genes related to human characteristics have been highlighted by some skeptics of data-driven approaches to scientific research. They have pointed out that the strength of the hypothesis-testing approach lies in its provision of a rigorous criterion for rejecting a particular relationship [27]. In contrast, they note that data-driven approaches inevitably turn up many correlations that turn out to be spurious. This contrast between the higher rigor of hypothesis-testing and the greater likelihood of spurious association in data-driven discovery methods seems to me to be reasonable, but it should not be taken to mean that data-driven methods are either bad science or useful only when linked in tandem with hypothesis-testing approaches.

It follows from the perspective we adopted in the introductory section of this paper on the philosophy of science that scientific "verification" does not mean meeting a Cartesian criterion of certainty through a Popperian procedure of elimination of false propositions via crucial experiments. Instead, scientific verification means establishing a high probability that a stated relationship between two phenomena provides a useful description under a wide range of observational perspectives (ideally most or all perspectives, but the ideals are achievable in different degrees with different phenomena). "High" is defined as sufficient for the uses to which it is to be put *and *by having been tested using the available and feasible means of examination. Such an approach leaves one without the comfort of absolute cut-offs, but it still allows comparison of "more" and "less", and achieving the "more fully verified" position turns out to be sufficient for progress in knowledge. Nonetheless, even within that framework, a statistically significant association is not a sufficient criterion for claiming a discovery has been made.

In biological beings, the large number of components of the phenomena sampled means that spurious associations are inevitable. If one tests a thousand alleles, with a 95% statistical cut-off, one can expect to produce around 50 spurious associations. Given the three billion base-pairs in the human genome, the problem is inevitably daunting. It cannot be repaired simply by raising the statistical cut-off, because that entails the well-known statistical "power" problem, which involves the trade-off between setting a criterion that is "too low", which falsely identifies spurious associations, or setting a criterion "too high", which fails to identify real but relatively weaker or rarer associations (generally described as a trade-off between "Type I" and "Type II" error). While there are currently rationales for different modes of adjustment for "multiple testing", there are no definitive criteria, and what is reasonable will undoubtedly vary by the empirical qualities of the specific cases.

Several commentators have suggested that integrating the inductive approaches of data "mining" with hypothesis-driven experimentation can optimize the strengths of each approach [28-30]. That is, after one identifies a potential discovery via data driven methods, one proceeds to traditional hypothesis-testing approaches. In some instances this proves to be a sensible suggestion, but it should not be taken to mean that inductive approaches are mere preliminaries to the "real" scientific effort of hypothesis testing, because hypothesis-testing procedures are also subject to the problems raised by the similarity-based nature of the highly complex beings of the biological realm. As the arguments made by Kelley and Scott [31] and of Kell and Oliver [29] begin to suggest, the model of invariant laws constructed through crucial experiments based on quantitatively described hypotheses does not make up the bulk of biological research. Relatively few experiments in biology are definitive, and relatively little of the knowledge in biology takes the form of species-invariant equations – not because biology is "immature" but precisely because biological organisms diverge. The existence of a particular phenomenon in one species does not guarantee its existence in another, and different organisms have evolved differently precisely to take advantage of specific ecodynamics. A few quantitative laws may cross most or all species, but most of the information about biological organisms is necessarily non-universal. In physics, a single measurement was taken to be definitive with regard to the non-existence of ether, only two observations are routinely cited to support the claim of gravitational lensing, and a small and (surprisingly) indistinct set of measurements are used to support the claim to the speed of light in a vacuum and the time-variation effect of relativity.

The luxury of drawing universal results from singular experiments does not exist in biology. As soon as one finds a phenomenon in one organism (and then species), the search must begin to see *whether *and to what extent the same phenomenon occurs in other species. This vitiates the notion of a crucial experiment that can establish a generalization across the biological domain. Moreover, the variation within biological organisms means that answers to biologically-based hypotheses are almost always judged using population-based statistical approaches. These statistical approaches face the same problems of trade-offs between "Type I" and "Type II Erorr" ("power") that occur in data-driven approaches. In practice, even the issue of multiple testing also arises, and the problems are enhanced by the fact that the sample size needed to adjudicate a test are frequently much larger than any feasible sample [32] – in human disease research, in some cases a larger sample size is needed than the known cases of the disease. Because similarity-based relations infect hypothesis testing as well as data-driven research, the "solution" to the verifiability problem for data-driven research cannot be merely to link the data-driven procedures in tandem with experimentation. Although experimentation may contribute part of the complex verification process, another part is the development of a set of criteria that indicate the likelihood of an association based on the nature of biological associations.

This article suggests that a reasonable starting point for identifying such criteria is the careful examination of the specific features of similarity-based generalization processes as they have actually functioned in key biological findings in which biologists have a good deal of confidence. The case of the zinc finger proteins has provided one example, which suggests as criteria the identification of lumpy gradients of similarity, a form-function linkage, a range of generality, and an evolutionary locus. Current researchers are already taking some account of the issues herein identified [33,34]. We are suggesting that more systematic integration of these features into the criteria for publishing conclusions about biological causation might further improve the reliability of data-driven research results.

## Conclusion

The idea that biological categories are similarity-based is widely accepted at the level of practice. The failure to integrate that idea into the philosophy of biology as a general principle may have contributed to a variety of errant presumptions, especially false assumptions about the homogeneity of groups of human gene pools. Systematic integration of the similarity-based categorization may help to avoid wasteful and erroneous research projects, as well as spurring insights about how to better take advantage of all of the information available in biological data sets. More broadly applied, such a vision may also help integrate biological findings into our socio-cultural discussions in a fashion that accepts natural diversity rather than effaces it.

## Competing interests

The author(s) declare that they have no competing interests.
